# Clinically isolated syndrome in collagen diseases; approaches and treatments

**DOI:** 10.1186/ar3559

**Published:** 2012-02-09

**Authors:** Kazumasa Yokoyama, Nobutaka Hattori

**Affiliations:** 1 Department of Neurology, Juntendo University School of Medicine, Hongo, Tokyo, 113-8421, Japan

## Background

Acute isolated neurological syndromes, such as optic neuropathy or transverse myelopathy, may cause diagnostic problems since they can be the first presentations in a number of demyelinating disorders including multiple sclerosis (MS) and collagen diseases. However, clinical presentation and lesions evidenced by magnetic resonance imaging may be similar. Collagen disease coexists in demyelinating disorders and frequently various collagen disease related autoantibodies are positive in daily practice.

Hence, the algorithm to overcome these diagnostic and therapeutic issues should be clarified.

### B cell immunity in demyelinating disorders

In primary demyelinating disease, MS, a renewed interest in the role of humoral immunity in the pathophysiology has been investigated because oligoclonalIgG band in the CSF and increased intrathecalIgG synthesis are used as an auxiliary diagnosis measure. Moreover, in the secondary progressive MS, meningeal B-cell follicles are associated with early onset of the disease and severe cortical pathology. B cell but not plasma cell depletion therapy with single treatment by Rituximab in MS showed reduced inflammatory brain lesions and clinical relapses.

### Oligodendropathy and astrocytopathy in demyelinating disorders

Neuromyelitisoptica (NMO) was previously considered to be a variant of MS but is now recognized as an astrocytopathy and secondary demyelinating event mimicking MS characteristics occurring due to autoantibody mediated mechanisms. Advancement of molecular biology makes it possible to differentiate MS by measuring abnormal autoantibody to aquaporin 4 (water channel). Interestingly, collagen diseases coexist more frequently with NMO than with MS. B cell depletion therapy with Rituximab has showed the same benefits, although, plasma exchange therapy is more effective with NMO than with MS.

### TNF therapy and demyelinating event

A report indicates that adverse events such as the demyelinating lesion in the brain, optic neuritis, and neuropathy occurred after treatment with anti-TNF alpha therapy in collagen disease, and TNF antagonizing therapy showed worsening in a clinical trial with MS. Pathogenesis of these events such as primary or secondary demyelination are still in enigma.

In this presentation, I will decode the temporal and spatial demyelinating processes in collagen diseases and show practical approaches and treatments.
